# Chronic Ethanol Consumption Induces Osteopenia via Activation of Osteoblast Necroptosis

**DOI:** 10.1155/2021/3027954

**Published:** 2021-10-27

**Authors:** Man Guo, Yong-Li Huang, Qi Wu, Li Chai, Zong-Zhe Jiang, Yan Zeng, Sheng-Rong Wan, Xiao-Zhen Tan, Yang Long, Jun-Ling Gu, Fang-Yuan Teng, Yong Xu

**Affiliations:** ^1^Department of Endocrinology and Metabolism, The Affiliated Hospital of Southwest Medical University, Luzhou, Sichuan 646000, China; ^2^Sichuan Clinical Research Center for Nephropathy, Luzhou, Sichuan 646000, China; ^3^Cardiovascular and Metabolic Diseases Key Laboratory of Luzhou, Luzhou, Sichuan 646000, China; ^4^Department of Outpatient, The Affiliated Hospital of Southwest Medical University, Luzhou, Sichuan 646000, China; ^5^Department of Pathology, The Affiliated Hospital of Southwest Medical University, Luzhou, Sichuan 646000, China; ^6^Experimental Medicine Center, The Affiliated Hospital of Southwest Medical University, Luzhou, Sichuan 646000, China; ^7^Department of Endocrinology, Yibin Second People's Hospital, Yibin, Sichuan 644000, China

## Abstract

Chronic high-dose alcohol consumption impairs bone remodeling, reduces bone mass, and increases the risk of osteoporosis and bone fracture. However, the mechanisms underlying alcohol-induced osteoporosis are yet to be elucidated. In this study, we showed that excess intake of ethyl alcohol (EtOH) resulted in osteopenia and osteoblast necroptosis in mice that led to necrotic lesions and reduced osteogenic differentiation in bone marrow mesenchymal stem cells (BMMSCs). We found that EtOH treatment led to the activation of the RIPK1/RIPK3/MLKL signaling, resulting in increased osteoblast necroptosis and decreased osteogenic differentiation and bone formation both in vivo and in vitro. We further discovered that excessive EtOH treatment-induced osteoblast necroptosis might partly depend on reactive oxygen species (ROS) generation; concomitantly, ROS contributed to necroptosis of osteoblasts through a positive feedback loop involving RIPK1/RIPK3. In addition, blocking of the RIPK1/RIPK3/MLKL signaling by necrostatin-1 (Nec-1), a key inhibitor of RIPK1 kinase in the necroptosis pathway, or antioxidant N-acetylcysteine (NAC), an inhibitor of ROS, could decrease the activation of osteoblast necroptosis and ameliorate alcohol-induced osteopenia both in vivo and in vitro. Collectively, we demonstrated that chronic high-dose alcohol consumption induced osteopenia via osteoblast necroptosis and revealed that RIPK1 kinase may be a therapeutic target for alcohol-induced osteopenia.

## 1. Introduction

Chronic and excessive alcohol consumption has harmful effects on multiple organs, including the brain, heart, liver, and skeleton [1–4]. Chronic heavy alcohol consumption is recognized as a major cause of secondary osteoporosis and is positively associated with impaired bone remodeling, extensive bone loss, and fracture [5–7]. Multiple mechanisms are responsible for alcohol-induced osteoporosis (AOP), including a direct effect on bone formation and bone resorption [8, 9]. Ethyl alcohol (EtOH) intake inhibits bone marrow mesenchymal stem cell- (BMMSC-) derived osteoblast proliferation and enhances osteoclast differentiation via the RANKL/RANKL/OPG signaling pathway that promotes bone resorption in long-term alcohol users [10, 11]. The imbalanced osteo/adipogenic lineage commitment of BMMSCs is modulated via activation of the PI3K/AKT/mTOR signaling that results in downregulation of runt-related transcription factor 2 (Runx2) and upregulation of peroxisome proliferator-activated receptor *γ* (PPAR*γ*) [12–14]. The indirect action of chronic alcohol-induced bone loss may also be regulated by inducing oxidative stress [13, 15]. It has been reported that with alcohol exposure, increased levels of reactive oxygen species (ROS) may directly precede the production of TNF-*α* and other inflammatory cytokines in the bone marrow and contribute to bone formation decrease [16]. Furthermore, increased endothelial nitric oxide synthase (eNOS) and overexpressed inducible nitric oxide synthase (iNOS) in the bone may contribute to osteocyte apoptosis and necrosis [17].

Apoptosis was identified as the early stage in alcohol-mediated osteoporosis, specifically an increase in osteoblast apoptosis and osteocytes [18, 19], while necrosis occurs in succession following extensive apoptosis. Deficiency or inhibition of the aspartate-specific cysteine protease-8 (caspase-8) can switch cell death into a specific form of necrosis, resulting in necroptosis [20–22]. Necroptosis, having typical morphological characteristics of necrosis, can be induced by tumor necrosis factor-*α* (TNF-*α*), a factor associated with suicide (Fas, which binds to the ligand CD95L, known as FASL), TNF-related apoptosis-inducing ligand (TRAIL), and other death cytokines [23–25]. Receptor-interacting serine/threonine protein kinase 1 (RIPK1) and 3 (RIPK3) activate the mixed lineage kinase domain-like protein (MLKL), which then recruits the necrosome to localize on the cell membrane and causes cell necroptosis [26, 27]. Furthermore, necroptosis can be regulated and pharmacologically inhibited by necrostatin-1 (Nec-1), the first small-molecule inhibitor of RIPK1 kinase to be developed [28–30]. Nec-1 is a cellular protector and has been widely used to investigate the role of RIPK1 in multiple disease models, including neurodegenerative and cerebrovascular diseases [31–33], atherosclerosis [34], heart attack [35, 36], and liver, retinal, and renal injury [37, 38]. Nec-1 has been reported to inhibit the death of chondrocytes in cartilage injury and promote cartilage repair [39]. Treatment with Nec-1 also reduces osteocyte necrosis and increases bone formation in glucocorticoid-induced osteoporosis (GIOP) rats and ovariectomized rats that resulted in the kinase RIPK1-dependent necroptosis [40, 41]. In addition, the necroptosis signaling pathway is also found to be involved in the pathophysiology of osteosarcoma [42]. However, it remains unknown whether RIPK1 mediates damage via activation of necroptosis in an alcohol-induced osteopenia model and the effect of necrostatin-1 on osteopenia requires further elucidation.

In the current study, we investigated the effect of ethanol treatment on osteogenesis and osteopenia and the underlying mechanisms that mediate necroptosis in alcohol-induced osteopenia. Our investigations revealed that EtOH treatment increased osteoblast necroptosis and decreased osteogenic differentiation and bone formation in vivo; ROS have been considered, at least in part, as a critical driving force for necroptosis; necrostatin-1 and N-acetylcysteine (NAC) ameliorated osteopenia via inhibition of the RIPK1/RIPK3/MLKL signaling pathways. Therefore, RIPK1 kinase and targeted antioxidants may be therapeutic targets for alcohol-induced osteopenia.

## 2. Materials and Methods

### 2.1. Animals

All animal experimental protocols were approved by the Animal Research Center of Southwest Medical University (No.: 20210928-007) and performed in accordance with the policies of Chinese animal research committees. Male C57BL/6J mice at 8 weeks were purchased from the SPF (Beijing) Biotechnology Co., Ltd. (China, SCXK 2016-0002) and acclimatized for two weeks with temperature and humidity controlled at 21 ± 0.5°C and 55 ± 10%, respectively. Then, mice were randomly divided into a control vehicle-treated (control), necrostatin-1 drug control group (Nec-1), chronic high-dose alcohol group (EtOH), and chronic high-dose alcohol add on necrostatin-1 treatment group (EtOH+Nec-1) (*n* = 15/group). Mice were administered 0.2 ml/10 g/d of alcohol (gradually increase concentration to 30% *v*/*v*) via gavage [43, 44] and 1.65 mg/kg/d of necrostatin-1 (Sigma-Aldrich, St. Louis, USA) by intraperitoneal injection (ip) [40]; the control received the ddH_2_O and 1000 *μ*l/kg/d of 1‰ DMSO solution (Sigma-Aldrich, St. Louis, USA) in the same volume by the same route for 22 weeks.

### 2.2. Dual-Energy X-Ray Absorptiometry (DXA)

Mice were anesthetized with 1% pentobarbital sodium (50 mg/kg body weight, ip). The whole body, lumbar spine, and bilateral femur scans were analyzed using the region of interest (ROI) tools (GE Healthcare Lunar, Germany). Bone mineral density (BMD) was automatically calculated for each ROI scan. A quality assurance scan of the calibration block provided with the scanner was performed daily.

### 2.3. Micro-Computed Tomography Assay (Micro-CT)

The distal femurs were harvested, fixed in 4% paraformaldehyde (PFA; Sigma-Aldrich, St. Louis, USA), and placed in fresh 70% ethanol. The specimens were then scanned using a high-resolution *μ*CT scanner (SCANCO Medical AG, *μ*CT 50, Brüttiselen, Switzerland) with a voxel size of 15 *μ*m, an X-ray tube voltage of 70 kVp, a current of 200 *μ*A, and an integration time of 300 ms. The scanner special software (SCANCO Medical Evaluation) was used to locate and analyze the distal femurs. The volumes-of-interest (VOIs) of bone tissue were 29 × 29 × 29 *μ*m^3^, and a total of 1800–2000 layers of cancellous bone tissue were defined as the final ROI. The datasets were loaded onto the Amira 5.3.1 software (Visage Imaging, Berlin, Germany) for visualization and analysis. In addition, BMD, bone volume/total volume (BV/TV), trabecular thickness (Tb.Th), trabecular number (Tb.N), trabecular separation (Tb.Sp), cortical thickness (Ct.Th), and 3D reconstructions for each specimen were calculated using the Amira software with 245 analysis thresholds.

### 2.4. Histological and Immunofluorescent and Immunohistochemistry Staining

The femurs were fixed in 4% PFA for 24 h, decalcified with 10% ethylenediaminetetraacetic acid (EDTA, pH 7.6–7.8) for 40 days at room temperature, and embedded in paraffin. Sections of 2 *μ*m thickness were deparaffinized, dehydrated, and stained with hematoxylin and eosin (H&E) and TRACP staining for histological and histomorphometric analysis (Leica, Germany). Bone remodeling activity was evaluated by the ratio of osteoid surface to bone surface (OS/BS, %), osteoblast surface to bone surface (Ob.S/BS, %), and eroded surface to bone surface (ES/BS, %), according to the recommendations of the ASBMR Histomorphometry Nomenclature Committee [45]. Sections of 4 *μ*m thickness were subjected to immunofluorescent and immunohistochemistry staining, blocked with 10% goat serum (Gibco, USA), and incubated with Runx2- or RIPK1- or RIPK3- or cleaved caspase-3-specific antibodies (1 : 100; Abcam, Cambridge, UK) at 4°C overnight, followed by a fluorescent secondary antibody (1 : 200; Cell Signaling Technology, USA) for immunofluorescent staining; treated with secondary antibody, incubated with the streptavidin-horseradish peroxidase complex, visualized with diaminobenzidine, and counterstained with hematoxylin for immunohistochemistry staining. The trabecular bone structure parameters and area percentage and the percentage of Runx2-, RIPK1-, and RIPK3-positive cells were calculated using the Image-Pro Plus software.

### 2.5. Isolation and Culturing of Mouse BMMSCs

Mouse BMMSCs were isolated from the bilateral femoral marrow of six-week-old male C57BL/6J mice. Single-cell suspensions were cultured in alpha-minimum essential medium (*α*-MEM; Hyclone, Logan, UT, USA) containing 15% fetal bovine serum (FBS; Gibco, Grand Island, NY, USA), 1% penicillin/streptomycin (Invitrogen, Grand Island, NY, USA), 2 mM L-glutamine (Sigma-Aldrich, St. Louis, USA), and 55 *μ*M 2-mercaptoethanol (Sigma-Aldrich, St. Louis, USA) in a 5% CO_2_ humidified atmosphere at 37°C. Nonadherent cells were discarded after 48 h, and the adherent BMMSCs were cultured for 16 days until colonies are formed and then passaged once for further experimental use. The essential medium was changed every 2 days.

### 2.6. Flow Cytometry

Flow cytometry was used to test the surface markers of the isolated BMMSCs at passage three. Adherent cells were collected and incubated with a positive surface epitope profile with monoclonal antibodies for mouse CD105 and Sca1 and negative cell surface markers with CD34 and CD45 (FITC anti-mouse CD34, CD45, CD105, and Sca1 antibodies; BD Biosciences, USA) and then were identified by flow cytometry analysis (BD Biosciences, San Jose, CA, USA), and the data were analyzed with EPICS XL software.

### 2.7. Osteogenic Differentiation and Mineralization Assays

The flow cytometry was used to identify BMMSCs in all experiments between three and seven passages. After the BMMSCs cultured in essential medium reached 70% confluency, the medium was replaced with an osteogenic medium containing essential medium and supplemented with 2 mM *β*-glycerophosphate (Sigma-Aldrich, St. Louis, USA), 100 *μ*M L-ascorbic acid 2-phosphate (Sigma-Aldrich, St. Louis, USA), and 10 nM dexamethasone (Sigma-Aldrich, St. Louis, USA).

Identified BMMSCs (1 × 10^5^) and mouse preosteoblastic MC3T3-E1 cells purchased from American Type Culture Collection (ATCC, USA) were seeded in 6-well plates with the osteogenic medium for 7 days. Alkaline phosphatase (ALP) staining (Beyotime Institute of Biotechnology, Shanghai, China) was performed using a standard protocol after 7 days of induction in 24-well plates. Further, alizarin red staining (Beyotime Institute of Biotechnology, Shanghai, China) was performed after culturing in osteogenic medium for 28 days to detect mineralized nodule formation. Images were captured with a converted microscope (Olympus, Tokyo, Japan), and quantitative analysis was performed with the Image-Pro Plus software.

### 2.8. Cell Proliferation Assay

The effects of ethanol or necrostatin-1 on cell proliferation were evaluated using the Cell Counting Kit-8 assay (Beyotime Institute of Biotechnology, Shanghai, China). Briefly, BMMSCs and MC3T3-E1 (1 × 10^5^ cells/well) were seeded in 96-well plates and cultured in 100 *μ*l growth medium until the cells proliferated to about 30%, respectively. The cells were then starved for 6 h, and the medium was replaced with osteogenic differentiation induction medium, and different concentrations of alcohol were added at 24, 48, and 72 h, respectively. After alcohol stimulation, 10 *μ*l CCK-8 solution and 90 *μ*l fresh medium were added to each well, with 5 repeat wells, and incubated at 37°C for 2 h, according to the manufacturer's protocol. Cell proliferation was assessed by measuring the absorbance at a wavelength of 450 nm (Thermo Fisher Scientific, MA, USA), and the appropriate time and concentration of alcohol intervention were determined. The same method was followed for necrostatin-1 (50 *μ*mol/l).

### 2.9. Real-Time PCR Analysis

Cells were cultured in 6-well plates with osteogenic medium and treated with alcohol (150 mM) and necrostatin-1 (50 *μ*M) for 48 h to detect the gene expression of necroptosis markers (RIPK1, RIPK3, and MLKL), treated for 7 days to test the expression of Runx2, then extracted total RNA using TRIzol reagent (Qiagen, Valencia, CA, USA), and assessed the concentration and purity by a spectrophotometer. Reverse transcriptase reactions were completed by ReverTra Ace® qPCR RT Kit (Toyobo, Japan), and real-time PCR was performed using SYBR® Premix Ex Taq (2x) (Toyobo, Japan). Finally, the CT values were calculated in relation to *β*-actin CT values (RQ = −ΔΔCt). Primers were synthesized by Primer Premier 5.0 software, using Primer-BLAST to confirm the definition of primers in NCBI website, and synthesized by Shanghai Biotechnology Co., Ltd. (Supplementary Table S1). The same method also applied to examine the gene expression of mouse bone tissue.

### 2.10. Western Blot Analysis

BMMSCs were cultured and treated as described previously, and proteins were harvested with RIPA cell lysis buffer system (Cell Signaling Technology, USA) supplied with phosphatase inhibitors. Total protein concentrations were quantified by BCA protein assay kit (Beyotime Institute of Biotechnology, Shanghai, China). Equal amounts of proteins (20 *μ*g) were separated on 10% SDS-PAGE for protein electrophoresis and transferred to 0.2 *μ*m polyvinylidene fluoride (PVDF) membranes, which were blocked with 5% nonfat dry milk for 1 h at room temperature and incubated at 4°C overnight with primary antibodies to mouse Runx2, RIPK1, RIPK3, MLKL, phosphor-RIPK3 (p-RIPK3), phosphor-MLKL (p-MLKL), cleaved caspase-3, and *β*-actin (1 : 1000; Abcam, Cambridge, UK) and phosphor-RIPK1 (p-RIPK1; 1 : 1000; Cell Signaling Technology, USA). After washing three times in PBST and incubated for 1 hour with HRP-conjugated second antibody at room temperature (1 : 2000, Abcam, Cambridge, UK), immunoreactive protein was detected using enhanced chemiluminescence reagents (Millipore, USA) and immunoblots were quantified with ImageJ software. MC3T3-E1 were cultured with osteogenic differentiation induction medium and treated as described previously to verify EtOH-induced osteoblast necroptosis.

### 2.11. Double-Label Immunofluorescent Staining

After 48 h of intervention on confocal dishes, cells were fixed with 4% PFA for 30 min, permeabilized in 0.5% Triton X-100 for 10 min, and incubated with primary antibodies of anti-mouse RIPK1 (1 : 100; Abcam, Cambridge, UK) and anti-rabbit RIPK3 (1 : 100; Abcam, Cambridge, UK) at 4°C for overnight and then incubated with fluorescent goat anti-mouse and goat anti-rabbit secondary antibodies (1 : 200; Abcam, Cambridge, UK) for 4 hours at room temperature. After stained with DAPI, acquired confocal images by OLYMPUS inverted laser scanning confocal microscope (Olympus, Tokyo, Japan) and mounted the positive coexpression of RIPK1 and RIPK3 rates.

### 2.12. Transmission Electron Microscopy (TEM)

After EtOH treatment for 48 h, the specimens were fixed in 2.5% glutaraldehyde for 2 h and postfixed in 1% osmium tetroxide for 90 min at 4°C, dehydrated in ethanol, and embedded in epoxy resin. The cells were examined under a transmission electron microscope (H-7700, Hitachi, Japan).

### 2.13. Intracellular Reactive Oxygen Species Assay

After EtOH treatment for 48 h, the ROS generation of MC3T3-E1 was detected by a ROS detection kit (Beyotime Institute of Biotechnology, Shanghai, China). The fluorescence of 2′-7′-dihydrodichlorofluoroscein diacetate (DCFH-DA) was determined using a spectrofluorophotometer, and the mean fluorescence intensity (MFI) was analyzed by flow cytometry.

### 2.14. ELISA Assay

Peripheral blood serum was collected; serum alcohol concentration was measured with mouse EnzyChrom™ Ethanol Assay Kit (BioAssay Systems, USA). TNF-*α* level was analyzed using mouse tumor necrosis factor-*α* ELISA kit (Andy Gene, China) with mouse peripheral blood serum and cell culture medium, according to the manufacturer's instructions. BALP, C-terminal telopeptide of type I collagen (CTX-I), tartrate-resistant acid phosphatase 5b (TRACP 5b) levels, and proinflammatory cytokines (IL-1*β* and IL-6) were analyzed using mouse ELISA kit (Andy Gene, China) with mouse peripheral blood serum.

### 2.15. Statistical Analysis

All experimental data were expressed as the mean ± standard deviation (SD) from at least three independent experiments. Comparisons between the two groups were analyzed using independent two-tailed Student's *t*-test, and comparisons between multiple groups were accomplished using one-way ANOVA with the Bonferroni adjustment with SPSS 22.0 software (SPNN Inc., Chicago, IL, USA). *p* values < 0.05 were considered statistically significant.

## 3. Results

### 3.1. EtOH Treatment Leads to Osteogenic Differentiation Reduction and Osteopenia

**Figure 1:**
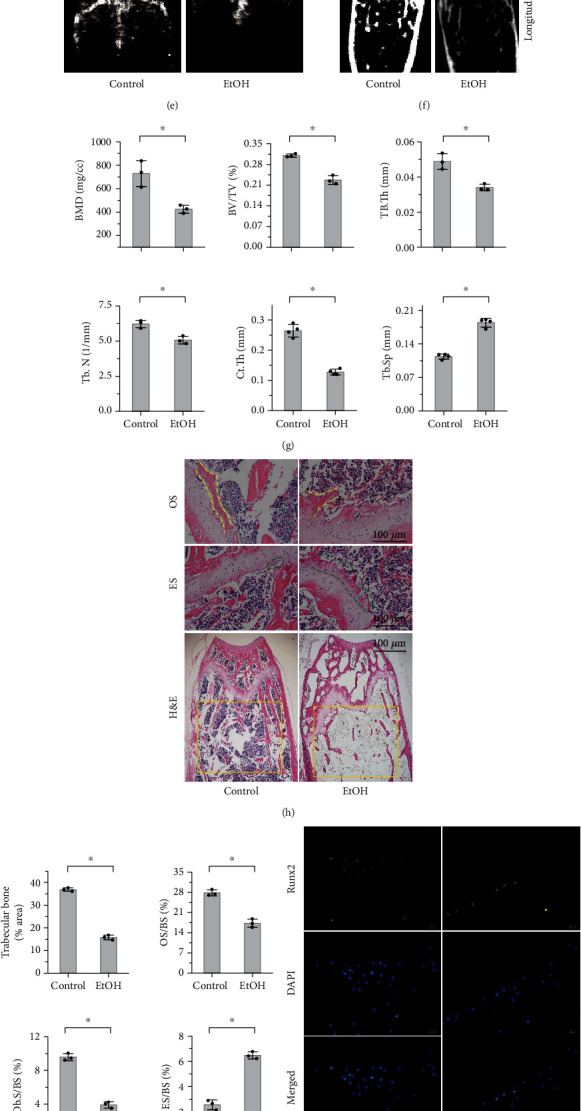
EtOH treatment leads to osteogenic differentiation reduction and osteopenia. (a) Body weight of mice for 22 weeks. (b) The serum alcohol concentration of the mice in the EtOH-treated group was twice as much as the control group when sacrificed. (c) The level of BALP in serum was decreased. (d, e) DXA analysis of mouse bilateral femurs (e) showed that chronic alcohol consumption significantly reduced the BMD of mice (d). (f, g) Micro-CT analysis of trabecular revealed that BMD, BV/TV, Tb.Th, Tb.N, and Ct.Th were markedly decreased in EtOH-treated mouse distal femurs and with the increase of Tb.Sp. (h, i) Histomorphometric analysis revealed that the distal trabecular bone structure was disordered and loose, the numbers of trabecular bone (h, yellow rectangle area), the ratio of OS/BS (h, two yellow dotted lines), and Ob.S/BS were significantly decreased, and the ratio of ES/BS (h, two green dotted lines) was increased compared to untreated mice (i). Bar: 100 *μ*m. (j–n) Both mRNA and protein levels of Runx2 in mouse bone tissue were significantly decreased that evaluated by immunofluorescent staining (j, k; bar: 100 *μ*m), western bolt analysis (l, m), and RT-PCR (n). All experimental data were verified in at least three independent experiments. Error bars represent the SD from the mean values. ^∗^*p* < 0.05. EtOH: ethyl alcohol; BALP: bone alkaline phosphatase; DXA: dual-energy X-ray absorptiometry; micro-CT: micro-computed tomography assay; BMD: bone mineral density; BV/TV: bone volume/total volume; Tb.Th: trabecular thickness; Tb.N: trabecular number; Ct.Th: cortical thickness; Tb.Sp: trabecular separation; OS: osteoid surface; ES: eroded surface; OS/BS: osteoid surface to bone surface; Ob.S/BS: osteoblast surface to bone surface; ES/BS: eroded surface to bone surface; Runx2: runt-related transcription factor 2.

In order to verify the effects of alcohol treatment on bone tissue, C57BL/6J male mice were administered via gavage with 0.2 ml/10 g/d (30% *v*/*v*) of alcohol for 22 weeks. All the animals were alive with no complications, and there was a significant difference in the body weight between the control group and EtOH-treated group after administration of alcohol for 18 weeks (*p* < 0.05, Figure 1(a)). The serum alcohol concentration of the mice in the EtOH-treated group was twice as much as the control group when sacrificed (*p* < 0.05), indicating the success of alcohol gavage (Figure 1(b)). BALP, an early marker of osteoblastic differentiation, in the serum was significantly decreased in the EtOH-treated group compared to that in the control group (*p* < 0.05, Figure 1(c)). Moreover, chronic alcohol consumption significantly reduced the bone mineral density (BMD) and mineral content of bilateral femurs, as measured by DXA (*p* < 0.05, Figures 1(d) and 1(e)), and markedly decreased the BMD, BV/TV, Tb.Th, Tb.N and Ct.Th, concomitantly, increased the Tb.Sp by micro-CT analysis (*p* < 0.05, Figures 1(f) and 1(g)). Histomorphometric analysis performed on the H&E stained sections revealed that the distal trabecular bone structure was disordered and loose, and the numbers of trabecular bone (yellow rectangle area, *p* < 0.01) and the ratio of OS/BS (two yellow dotted lines) and Ob.S/BS were significantly decreased (*p* < 0.05); the ratio of ES/BS (two green dotted lines) was increased (*p* < 0.01) compared to untreated mice (Figures 1(h) and 1(i)). Immunofluorescent staining, western blotting, and RT-PCR detected the expression of Runx2, a specific marker of osteogenic differentiation, and the results revealed that mRNA and protein levels of Runx2 were significantly decreased (Figures 1(j)–1(n)). Thus, this indicated that chronic alcohol consumption can lead to osteogenesis reduction and osteopenia in mice.

### 3.2. EtOH Treatment Leads to Osteoblast Necroptosis

**Figure 2:**
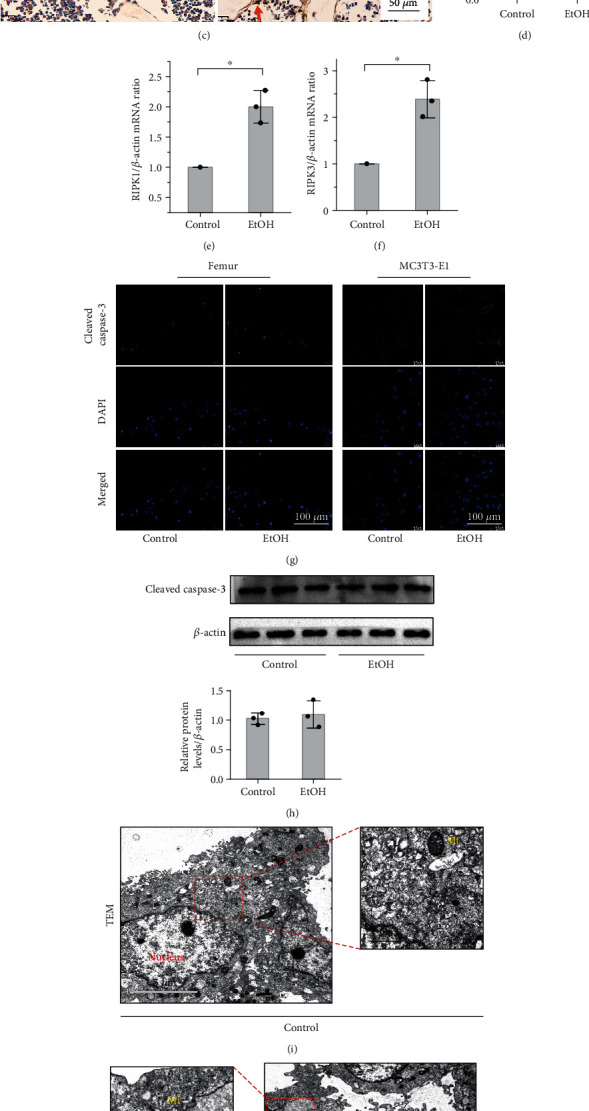
EtOH treatment leads to osteoblast necroptosis in vivo. (a–d) Immunohistochemistry (IHC) staining to evaluate the differences in RIPK1 and RIPK3 expression and identify osteoblasts according to the morphology and distribution in bone tissue, finally located the expression of RIPK1 and RIPK3 on osteoblasts. The number of RIPK1-positive (a, b) and RIPK3-positive (c, d) osteoblasts markedly increased in femurs with EtOH treatment. The red arrow indicates the positive expression in osteoblasts. Bar: 50 *μ*m. (e, f) Real-time PCR showed upregulated in the mRNA expression of RIPK1 and RIPK3 with EtOH treatment. Immunofluorescent staining of femurs and MC3T3-E1 cells (g) and western blot of mouse bone tissue (h) showed that the protein levels of cleaved caspase-3 were increased in the EtOH-treated group, but the difference was not statistically significant (*p* > 0.05). Bar: 100 *μ*m. TEM analysis revealed that cells exhibited normal nuclear and cytoplasmic morphology in the control group (i) and displayed typical necrotic ultrastructural changes including a disrupted plasma membrane, fragmented and vacuolated mitochondrial membranes (yellow mark), and overflowed cytoplasmic content after exposure to 150 mM EtOH for 48 h (j). Bar: 5 *μ*m and 1 *μ*m, respectively. Western blotting showed upregulated in the protein expression of RIPK1 and p-RIPK1 (k, l) and RIPK3 and p-RIPK3 (m, n) with EtOH treatment in vivo. Error bars represent the SD from the mean values. ^∗^*p* < 0.05. RIPK1: receptor-interacting serine/threonine kinases 1; RIPK3: receptor-interacting serine/threonine kinases 3; TEM: transmission electron microscopy; Mt: mitochondrion.

To investigate the cause of EtOH-induced osteogenesis reduction and osteopenia, we detected necroptosis signaling. Interestingly, we found that high doses of EtOH markedly elevated the osteoblast necroptosis of bone tissue, as located and evidenced by the increased number of RIPK1-positive cells (Figures 2(a) and 2(b)) and RIPK3-positive cells (Figures 2(c) and 2(d)). The mRNA levels of RIPK1 and RIPK3 were correspondingly increased (Figures 2(e) and 2(f)). The levels of bone resorption markers, CTX-I and TRACP 5b, in serum (*p* < 0.05, Supplementary Figure S1a, b) and the number of osteoclasts in the femurs identified by TRACP staining increased in the EtOH-treated mice. Surprisingly, the expression of RIPK1 in osteoclasts had no significant difference between the alcohol intervention group and the control group with colocalization (Supplementary Figure S1c). Overall, necroptosis of osteoblasts was more obvious than osteoclasts with alcohol intervention, so our experiment mainly focused on bone formation rather than bone resorption.

**Figure 3:**
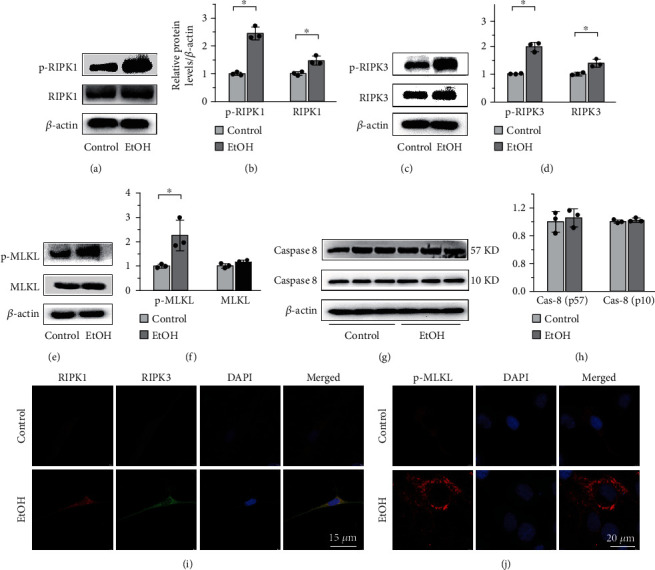
EtOH induces osteoblast necroptosis by activating the RIPK1/RIPK3/MLKL signaling in vitro. Western blot showed that the expressions of RIPK1 and p-RIPK1 (a, b), RIPK3 and p-RIPK3 (c, d), and p-MLKL (e, f) on MC3T3-E1 cells were elevated in the EtOH treatment group, while the expression of caspase-8 was not significantly influenced (g, h). Double-labeled immunofluorescence staining confirmed that necroptosis makers RIPK1 and RIPK3 coexpressed in the EtOH-treated MC3T3-E1 cells (i). Bar: 15 *μ*m. And p-MLKL aggregated on the cell membrane, leading to perforation and destruction of the integrity of proliferating MC3T3-E1 osteoblasts (j). Bar: 20 *μ*m. Error bars represent the SD from the mean values. ^∗^*p* < 0.05. p-MLKL: phosphorylated mixed lineage kinase domain-like protein.

To determine the type of cell death that a cell undergoes, antiactive caspase-3 was detected. The results showed that the protein levels of cleaved caspase-3 in mouse bone tissue and MC3T3-E1 cells were increased in the EtOH-treated group evaluated by immunofluorescent staining and western bolt analysis, but the difference was not statistically significant (*p* > 0.05, Figures 2(g) and 2(h)). Consistently, TEM analysis revealed that cells displayed typical necrotic ultrastructural changes including a disrupted plasma membrane, fragmented and vacuolated mitochondrial membranes, and overflowed cytoplasmic content after exposure to 150 mM EtOH for 48 h (Figures 3(i) and 3(j)) that exhibited the typical characteristic of necroptosis rather than apoptosis. The above results suggested that apoptosis accounts for a small proportion in the occurrence of alcohol-induced osteopenia, but mainly necroptosis.

Further, to confirm the activation of necroptosis in the EtOH-treated group, we performed western blotting to semiquantitatively analyze the changes in the protein expression levels. The results showed upregulated expression of RIPK1 and p-RIPK1 (Figures 2(k) and 2(l)) and RIPK3 and p-RIPK3 (Figures 2(m) and 2(n)) with EtOH treatment in vivo, indicating that chronic heavy alcohol-induced bone loss in mice was associated with osteoblast necroptosis activation.

### 3.3. EtOH Induces Osteoblast Necroptosis by Activating the RIPK1/RIPK3/MLKL Signaling

To investigate the mechanism of EtOH-induced osteoblast necroptosis, we added 150 mM EtOH to BMMSCs, which were identified by flow cytometry analysis (Supplementary Figure S2a) and then induced to differentiate into osteoblasts that were evaluated by alkaline phosphatase (ALP) staining (Supplementary Figure S2b) and alizarin red staining (Supplementary Figure S2c), for 48 h using the CCK-8 assay (Supplementary Figure S3a, b). Quantitative western blotting showed that EtOH treatment elevated the expression of RIPK1 and p-RIPK1 (Figures 3(a) and 3(b)), RIPK3 and p-RIPK3 (Figures 3(c) and 3(d)), and p-MLKL (Figures 3(e) and 3(f)), while the expression of caspase-8 was not significantly influenced (Figures 3(g) and 3(h)). Double-labeled immunofluorescence staining confirmed that MC3T3-E1 significantly coexpressed the necroptosis markers RIPK1 and RIPK3 after EtOH treatment (Figure 3(i)). Confocal microscopy further proved that p-MLKL aggregated on the cell membrane, leading to perforation and destruction of the integrity of proliferating MC3T3-E1 (Figure 3(j)). Therefore, we concluded that alcohol induced osteoblast necroptosis by activating the RIPK1/RIPK3/MLKL signaling.

### 3.4. Necrostatin-1 Treatment Ameliorates Osteopenia and Osteogenic Differentiation Reduction in EtOH-Treated Mice

**Figure 4:**
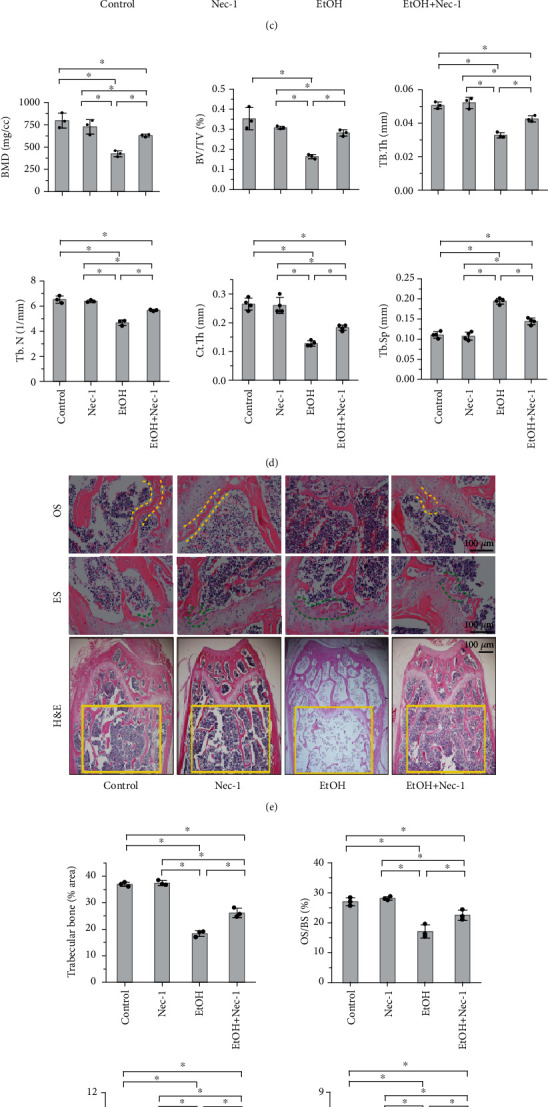
Necrostatin-1 treatment ameliorates osteopenia in EtOH-treated mice. Nec-1 treatment ameliorated EtOH-induced low BMD in femurs (a-d) and BV/TV, Tb.Th, Tb.N, and Ct.Th in the distal femurs, as assessed by DXA (a, b) and axial micro-CT cross sections and 3D reconstructions (c, d), respectively. H&E stained sections revealed that Nec-1 treatment inhibited the reduction in trabecular bone volume and number and facilitated partial recovery of the destroyed trabecular structure (e, yellow rectangle area), increased the ratio of OS/BS (e, two yellow dotted lines) and Ob.S/BS, and decreased the ratio of ES/BS (e, two green dotted lines) compared to EtOH-treated mice (f) Bar: 100 *μ*m. Nec-1 treatment significantly increased bone formation, as indicated by the increase in alkaline phosphatase activity (ALP staining) and the formation of mineralized nodules (alizarin red staining) in osteoblasts (g) and serum alkaline phosphatase activity in mice (h). Error bars represent the SD from the mean values. ^∗^*p* < 0.05. Nec-1: necrostatin-1.

**Figure 5:**
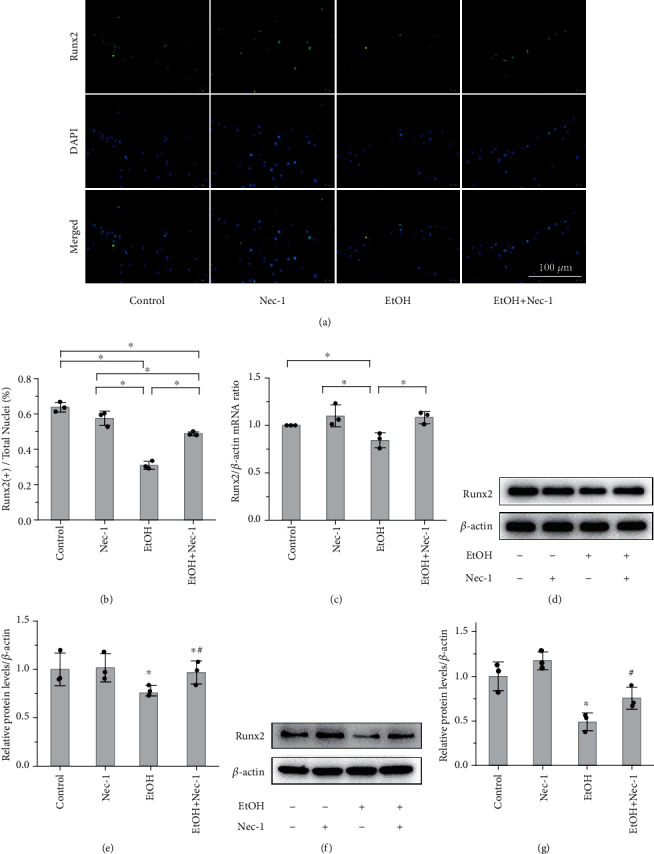
Necrostatin-1 treatment ameliorates osteogenic differentiation reduction. Immunofluorescence staining (a, b; bar: 100 *μ*m), RT-PCR (c), and western blot analysis both in vivo (d, e) and in vitro (f, g) showed that the expression level of Runx2 was downregulated after EtOH treatment; however, necrostatin-1 treatment upregulated the expression level. (a–e) Data from mice. (f, g) Osteoblasts. Error bars represent the SD from the mean values. ^∗^*p* < 0.05 compared with the control group. ^#^*p* < 0.05 compared with the EtOH-treated group.

Next, to examine whether necrostatin-1 treatment ameliorated osteopenia in EtOH-treated mice, we treated the mice with 1.65 mg/kg/d of Nec-1 for 22 weeks and BMMSCs with 50 *μ*M of Nec-1 for 48 h, respectively (Supplementary Figure S3c, d). We found that Nec-1 treatment ameliorated EtOH-induced low BMD, trabecular BV/TV, Tb.Th, Tb.N, and Ct.Th in the distal femurs, as assessed by DXA, micro-CT cross sections, and 3D reconstructions, respectively (Figures 4(a)–4(d)). Further, H&E staining revealed that Nec-1 treatment inhibited the reduction in trabecular bone volume and number, facilitated partial recovery of the loose trabecular structure, and increased the ratio of OS/BS and Ob.S/BS compared to EtOH-treated mice (Figures 4(e) and 4(f)). Moreover, Nec-1 treatment significantly increased bone formation, as indicated by the increase in alkaline phosphatase activity and the formation of mineralized nodules in osteoblasts (Figure 4(g)) and serum ALP activity in mice (Figure 4(h)). Further, immunofluorescence staining (Figures 5(a) and 5(b)), qPCR (Figure 5(c)), and western blot analysis both in vivo (Figures 5(d) and 5(e)) and in vitro (Figures 5(f) and 5(g)) showed that the expression level of Runx2 was downregulated after EtOH treatment; notably, necrostatin-1 treatment upregulated these expression levels.

### 3.5. Necrostatin-1 and NAC Treatment Ameliorates Osteopenia by Inhibiting the RIPK1/RIPK3/MLKL Signaling

**Figure 6:**
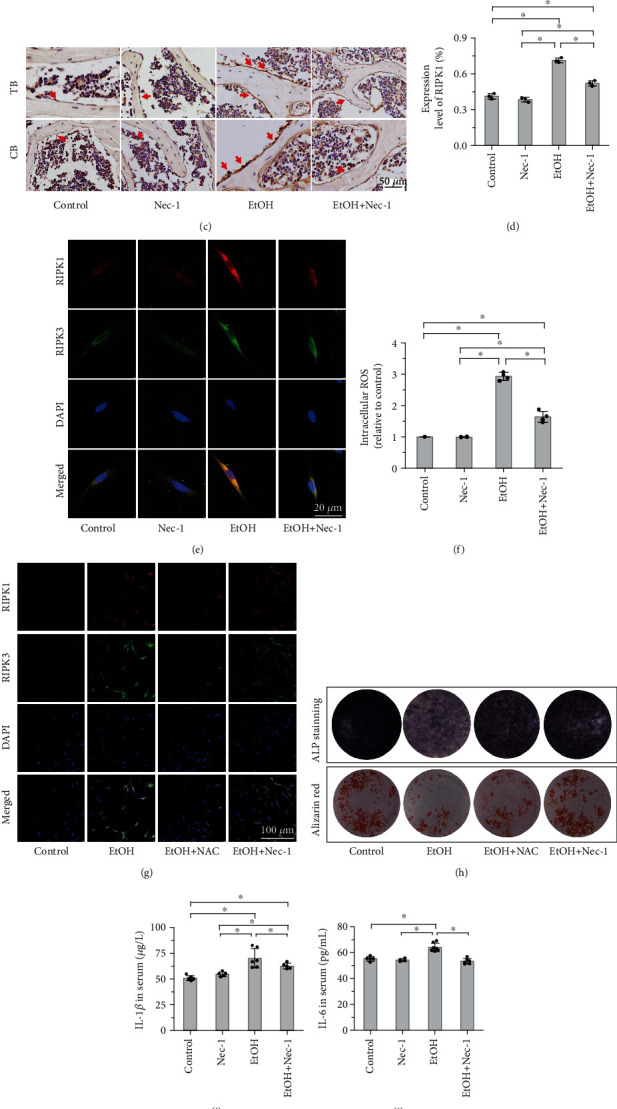
Necrostatin-1 and NAC treatment inhibited the RIPK1/RIPK3/MLKL signaling. (a, b) Western blot analysis showed that EtOH treatment activated the expression levels of the RIPK1 signaling pathway, including p-RIPK1, p-RIPK3, and p-MLKL compared to control mice and MC3T3-E1 cells, and necrostatin-1 treatment lowered the elevated levels both in vivo (a) and in vitro (b). (c, d) Immunohistochemistry staining showed that the expression of RIPK1 in bone tissue increased with EtOH treatment and inhibited by necrostatin-1. The red arrow indicates the positive expression in osteoblasts. Bar: 50 *μ*m. Confocal laser scanning microscopy revealed that Nec-1 treatment alleviated the increase of RIPK1- and RIPK3-positive osteoblasts in vitro (e). Bar: 20 *μ*m. (f-h) High dose of EtOH consumption increased the intracellular ROS levels compared with the control group, and Nec-1 directly inhibited the elevated ROS by flow cytometry. The addition of NAC effectively reduced EtOH treatment necroptosis of the osteoblastic cells as NAC attenuated the upregulation of RIPK1 and RIPK3 (g). NAC treatment significantly increased bone formation, as indicated by the increase in ALP staining and the formation of mineralized nodules (alizarin red staining) in osteoblasts (h). (i, j) The levels of serum proinflammatory cytokines (IL-1*β* and IL-6) were significantly downregulated by Nec-1 treatment compared to the EtOH-treated mice. The level of TNF-*α* elevated in mouse serum (k) and osteoblast culture medium (l) after EtOH treatment by ELISA assay, but had no significant difference after Nec-1 treatment. Error bars represent the SD from the mean values. ^∗^*p* < 0.05 compared with the control group. ^#^*p* < 0.05 compared with EtOH treatment. TB: trabecular bone; CB: cortical bone; ROS: reactive oxygen species; NAC: N-acetylcysteine; TNF-*α*: tumor necrosis factor-*α*.

We further explored whether necrostatin-1 treatment could inhibit RIPK1 kinase and investigated its effects on the downstream signaling pathway of RIPK1 with EtOH intervention. Western blot analysis showed that Nec-1 treatment lowered the elevated levels of p-RIPK1, p-RIPK3, and p-MLKL both in vivo (Figure 6(a)) and in vitro (Figure 6(b)). These results were confirmed by RT-PCR analysis, as shown in Supplementary Figure S4a–c. In the alcohol mouse model, the increased expression of RIPK1 in osteoblasts was significantly inhibited by Nec-1 treatment, as located and observed by immunohistochemistry staining (Figures 6(c) and 6(d)). In addition, confocal laser scanning microscopy revealed that Nec-1 treatment alleviated the increase of RIPK1- and RIPK3-positive MC3T3-E1 in vitro (Figure 6(e)). These results suggest that necrostatin-1 treatment ameliorates osteopenia in EtOH-treated mice by inhibiting the RIPK1/RIPK3/MLKL signaling.

Oxidative stress is known as one trigger for alcohol-induced osteopenia, and increased levels of ROS may directly precede the production of inflammatory cytokines in the bone marrow and contribute to decreased bone formation [16]. However, whether ROS could increase osteoblast necroptosis in alcohol-induced osteopenia remains unclear. Therefore, we measured the effect of alcohol intervention on the intracellular ROS levels by flow cytometry, and our results supported that high dose of EtOH consumption increased the intracellular ROS levels compared with the control group, and Nec-1 directly inhibited the elevated ROS (Figure 6(f)). Several necroptotic stimuli have been proposed to induce necroptosis, and ROS are essential factors [46]. Next, we explored whether ROS are important for EtOH-induced necroptosis in osteoblastic cells, using the antioxidant NAC to inhibit the production of ROS. In this study, the addition of NAC effectively reduced EtOH treatment necroptosis of the osteoblastic cells as NAC attenuated the upregulation of RIPK1 and RIPK3 (Figure 6(g)). Meanwhile, NAC treatment significantly increased bone formation, as indicated by the increase in ALP staining and the formation of mineralized nodules (alizarin red staining) in osteoblasts (Figure 6(h)). Together, these results highlight that osteoblast necroptosis, at least in part, promotes the generation of ROS, and on the other hand, ROS play a critical role in the regulation of excessive EtOH treatment-induced osteoblast necroptosis via a positive feedback loop.

## 4. Discussion

Chronic and excessive alcohol consumption causes a reduction in bone mass and destruction of the bone microstructure due to an impairment and/or imbalance in bone remodeling. However, the underlying mechanisms have not yet been elucidated. In this study, we successfully established an alcohol-induced osteopenia mouse model and found that osteopenia is accompanied by decreased bone formation and increased osteoblast necrosis, which was reported in studies on GIOP and ovariectomized rats [40, 41, 47]. Long-term detrimental effects of alcoholism have toxic effects on BMMSCs and affect their differentiation into osteoblasts by downregulating the expression of Runx2, which in turn leads to low bone mass [48, 49]. In addition, previous studies have confirmed that apoptosis was involved in alcohol-induced osteoporosis [18, 50, 51]. Herein, we found that loose trabeculae were more obvious, and the lacunae volume was larger in the EtOH-treated mice than in the control mice. Moreover, some of the MC3T3-E1 treated with alcohol suffered a necrotic change as observed from certain morphological changes and specific markers. These conclusions indicate the involvement of necrosis-associated mechanisms in the osteoblasts treated with EtOH.

Necroptosis is a caspase-independent form of programmed cell death that is triggered by ligation of death receptors or initiators that bind deubiquitylated RIPK1 to autoactivated RIPK3 and initiate the phosphorylation of a pseudokinase substrate MLKL, which then combines with p-RIPK3 to form a necrosome and translocates to the membrane to promote cell lysis [46, 52, 53]. RIPK1 and RIPK3 are key necroptotic effectors that mediate the necroptosis pathway, phosphorylated MLKL is involved in the execution of necroptotic cell death, and caspase-8 is a key factor regulating apoptosis or necroptosis [54]. In this study, we found that the RIPK1/RIPK3/MLKL pathway was involved in alcohol-induced bone disorders and osteoblast necroptosis both in vivo and in vitro. However, we found that the expression of caspase-8 was not significantly downregulated or inhibited, and the protein levels of cleaved caspase-3, a specific marker of apoptosis, increased in the EtOH-treated group without statistically significant difference. Thus, both necroptosis and apoptosis contributed to alcohol-mediated osteopenia, and necroptosis performed the main inducement. The combination of necroptosis and apoptosis also occurred simultaneously in the process of dexamethasone-induced MC3T3-E1 cell death [55] and affected the outcomes in liver diseases [56] and other inflammation or tissue damage [57–59].

Studies have revealed important functions and regulatory mechanisms of RIPK1 in inflammation and have indicated the pathological roles of RIPK1 in many human diseases [60]. For noninfectious diseases, inhibition of the RIPK1-dependent necroptosis pathway can ameliorate ischemia-reperfusion injury in the brain, kidney, and heart [61] and alleviate retinal degeneration, brain impact trauma, ethanol-induced liver injury, multiple sclerosis, and tumor progression [37, 62]. Others, TNF-induced systemic inflammatory response syndrome, and even metastasis in cancer cells were also closely related to RIPK1 kinase activation [63]. For infectious diseases, not only virus infections including influenza and HIV but also bacterial infections cause necroptosis-mediated inflammatory responses [64]. It indicated RIPK1 kinase may be a therapeutic target for multiple human diseases. However, it remains unclear whether blocking RIPK1 kinase by necrostatin-1 could repair the damage caused by alcohol-induced osteopenia. Therefore, we administered necrostatin-1 to EtOH-treated mice, BMMSCs, and MC3T3-E1, which can specifically target RIPK1 kinase and block the formation of the necrosome. It is reported that necrostatin-1 suppresses cell death triggered by caspase inhibition and has no effect on apoptosis [29, 65]. Our research suggested that necrostatin-1 significantly reduced necrosis of osteoblast induced by alcohol as well as the decreased expression of necroptosis markers RIPK1, RIPK3, and MLKL and blocked/reduced inflammation release as detected by the proinflammatory cytokines (IL-1*β* and IL-6) in mouse serum (Figures 6(i) and 6(j)). The thinned trabecular recovered partially, the levels and capability of osteogenic differentiation from BMMSCs improved, and the expression of the bone formation markers significantly increased after necrostatin-1 treatment. These results suggest that necrostatin-1 directly inhibits the expression of RIPK1, affects the activation of osteoblast necroptosis, and promotes the survival and proliferation of BMMSCs in the presence of death receptor ligands [28], eventually leading to a reduction in osteoblast necrosis and increased bone formation. Consequently, we speculate that necroptosis may be partly involved in the pathological processes underlying alcohol-induced osteopenia and that RIPK1 kinase may be an effective therapeutic target for alcohol-induced osteopenia [40].

Studies revealed that necroptosis was involved in the regulated cell death induced by TNF-*α* in nonalcoholic fatty liver disease [66], osteoblasts [67], glycerol-induced acute kidney injury [68], etc. However, the specific regulatory mechanism of TNF-*α* on bone cells and the molecular mechanism in the pathogenesis of osteoporosis remain unclear. It has been reported that TNF-*α* plays a role in promoting alcohol-induced osteoporosis [69, 70]. Given that necroptosis is a special type of necrosis mainly mediated by the stimulating death receptor of TNF-*α* [27, 71], we examined the level of TNF-*α* in mouse serum and osteoblastic culture medium after EtOH intervention and Nec-1 treatment by ELISA. Both serum and medium contained elevated levels of TNF-*α* in the EtOH-treated mice and cells but had no significant difference after Nec-1 treatment (Figures 6(k) and 6(l)). Therefore, we conjectured that alcohol could induce necroptosis by activating the expression of TNF-*α* and downstream molecules. Unfortunately, we could not prove the role of TNF-*α* in the process of EtOH-induced necroptosis. Moreover, detecting the RIPK1/RIPK3/MLKL axis by treating osteoblasts with TNF-*α* plus the specific inhibitor of necroptosis with or without Nec-1 is necessary to validate that chronic alcohol conditions promote RIPK1 activation with TNF signaling.

ROS have been considered as a driving force for necroptosis, the mitochondrial ROS derived in response to the TNF/TNFR1 pathway [72], and nonmitochondrial ROS generated via TNF-induced necroptosis [73]. Studies showed that treatment with TNF-*α* could stimulate ROS production, which enhanced necrosome formation [74]. ROS promote RIPK1 autophosphorylation on serine residue 161 (S161), and three crucial cysteines (cysteines 257, 268, and 586) in RIPK1 are required for sensing ROS to regulate RIPK1. The major function of RIPK1 kinase activity in TNF-induced necroptosis is to autophosphorylate S161 which then forms a functional necrosome [75]. TNF-induced ROS function in a positive feedback to enhance necrosome formation and induce necroptosis, when the necrosome complex was impaired or knockdown the RIPK1 or RIPK3, led to a decrease in ROS production [74]. In our study, alcohol treatment increased the production of intracellular ROS levels and necrosome complex. Conversely, treatment with Nec-1 markedly suppressed necroptosis and ROS production in EtOH intervention osteoblasts. Altogether, osteoblast necroptosis partly promotes the generation of ROS; on the other hand, ROS contributed to necroptosis of osteoblasts through a positive feedback loop involving RIPK1/RIPK3.

**Figure 7:**
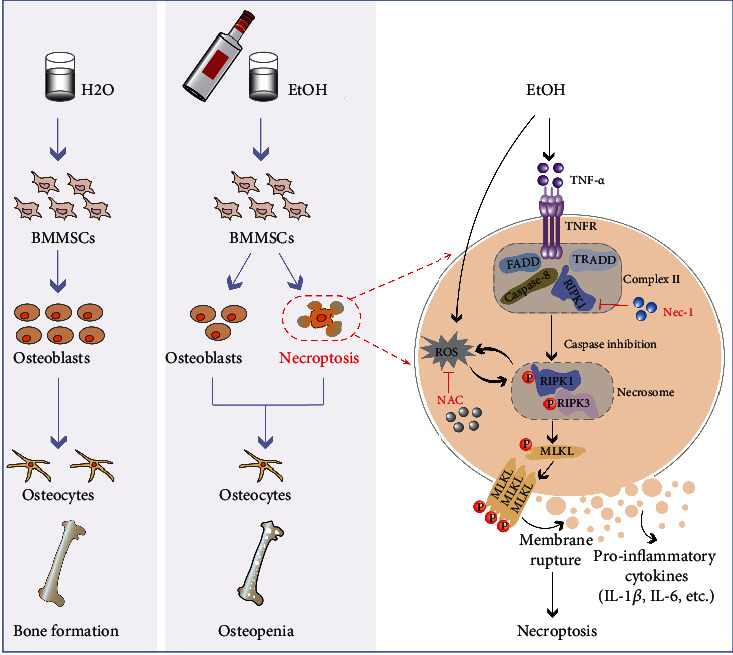
EtOH treatment induced osteopenia by increasing osteoblast necroptosis and decreasing BMMSC osteogenic differentiation. Chronic EtOH consumption results in osteopenia and osteoblast necroptosis in mouse model and leads to a necrotic morphology lesion and osteogenic differentiation reduction in bone marrow mesenchymal stem cells (BMMSCs). The ligation is stimulated by EtOH treatment that binds deubiquitylated RIPK1 to autoactivated RIPK3 and initiates the phosphorylation of a pseudokinase substrate MLKL, which then combines with p-RIPK3 to form a necrosome and translocates to the membrane to promote cell lysis and inflammation release (IL-1*β* and IL-6). Excessive EtOH treatment-induced osteoblast necroptosis might partly depend on ROS generation; concomitantly, ROS contributed to necroptosis of osteoblasts through a positive feedback loop involving RIPK1/RIPK3. Nec-1 and NAC inhibit the expression of RIPK1, affect the activation of necroptosis, and promote the survival and proliferation of osteoblast in the presence of death receptor ligands, eventually leading to a reduction in osteoblast necrosis and increased bone formation. TNFR: tumor necrosis factor receptor; FADD: Fas-associated protein with death domain; TRADD: TNFR1-associated death domain.

We first demonstrated that the necroptotic pathway underlies alcohol-induced osteopenia and provides evidence to explore a new therapeutic target in mice (Figure 7). Necrostatin-1 treatment successfully treated osteopenia and had long-term therapeutic inhibition in mice due to chronic heavy alcohol consumption. Generally speaking, necrostatin-1 is relatively safe from the current animal experimental observation, but the therapeutic safety and effect of necrostatin-1 on humans require investigation in future studies, including preclinical tests and clinical trials. Other programmed cell deaths, such as apoptosis, autophagy, and pyroptosis, play crucial roles in metabolic processes. Exploring the molecular mechanism in the pathogenesis of AOP, developing selective, potent, and safe biomarkers or inhibitors to measure and treat alcohol-induced osteoblast death remains a key challenge in clinical development.

## 5. Conclusions

In summary, this study provides evidence that excessive EtOH treatment leads to activation of the RIPK1/RIPK3/MLKL signaling, promotes the generation of ROS, resulting in an increase in osteoblast necroptosis and a decrease in BMMSC osteogenic differentiation both in vivo and in vitro, and causes bone mass reduction. Necrostatin-1-mediated treatment effectively inhibits the RIPK1/RIPK3/MLKL-dependent signaling, and NAC partly prevents the production of ROS and decreases the activation of necroptosis through a positive feedback loop involving RIPK1/RIPK3 and finally ameliorates alcohol-induced osteopenia by reducing osteoblast necroptosis. Collectively, we demonstrated that chronic high-dose alcohol consumption induces osteopenia via necroptosis and thereby discovered that RIPK1 kinase may be a therapeutic target for alcohol-induced osteopenia. However, the detailed mechanism and other molecules involved need to be further investigated.

## Data Availability

The original data used to support the findings of this study are available from the corresponding authors upon request.

## Supplementary Materials

Supplementary Materials Table S1: DNA primers used in quantitative real-time PCR. Figure S1: bone resorption in alcohol-induced osteopenia. Figure S2: identified the isolated mouse BMMSCs and osteoblast. Figure S3: CCK-8 assay was performed to determine the cell proliferation ability. Figure S4: necrostatin-1 treatment inhibited the RIPK1/RIPK3/MLKL signaling.
