# Supplementary material for: Chronic Ethanol Consumption Induces Osteopenia via Activation of Osteoblast Necroptosis
Source: Oxid Med Cell Longev. 2021 Oct 27;2021:3027954. doi: 10.1155/2021/3027954 (PMC8566044; doi:10.1155/2021/3027954)
Supplement: Supplementary Materials — Table S1: DNA primers used in quantitative real-time PCR. Figure S1: bone resorption in alcohol-induced osteopenia. Figure S2: identified the isolated mouse BMMSCs and osteoblast. Figure S3: CCK-8 assay was performed to determine the cell proliferation ability. Figure S4: necrostatin-1 treatment inhibited the RIPK1/RIPK3/MLKL signaling. [file 3027954.f1.docx]

**Chronic ethanol consumption induces osteopenia via activation of**

**osteoblast necroptosis**

Man Guo^1,2,3, #^, Yong-Li Huang^4, #^, Qi Wu^5, #^, Li Chai^5^, Zong-Zhe Jiang^2,3,6^, Yan Zeng^1,2,3^, Sheng-Rong Wan^1,2,3^, Xiao-Zhen Tan^2,3,6^, Yang Long^2,3,6^, Junling Gu^7^, Fang-Yuan Teng^2,3,6,^ *, Yong Xu^1,2,3,^ *

^1^ Department of Endocrinology and Metabolism, the Affiliated Hospital of Southwest Medical University, Luzhou, Sichuan 646000, China

^2^ Sichuan Clinical Research Center for Nephropathy, Luzhou, Sichuan 646000, China

^3^ Cardiovascular and Metabolic Diseases Key Laboratory of Luzhou, Luzhou, Sichuan 646000, China

^4^ Department of Outpatient, the Affiliated Hospital of Southwest Medical University, Luzhou, Sichuan 646000, China

^5^ Department of Pathology, the Affiliated Hospital of Southwest Medical University, Luzhou, Sichuan 646000, China

^6^ Experimental Medicine Center, the Affiliated Hospital of Southwest Medical University, Luzhou, Sichuan 646000, China

^7^ Department of Endocrinology, Yibin Second People's Hospital, Yibin, Sichuan 644000,China

**Supplemental materials**

**TABLE. S1** DNA primers used in quantitative real time PCR.

**FIGURE S1** Bone resorption in alcohol-induced osteopenia.

**FIGURE S2** Identified the isolated mouse BMMSCs and osteoblast.

**FIGURE S3** CCK-8 assay was performed to determine the cell proliferation ability.

**FIGURE S4** Necrostatin-1 treatment inhibited the RIPK1/RIPK3/MLKL signaling.

**TABLE S1** DNA primers used in quantitative real time PCR.

| Primer name | sequence (5**′-** 3**′**) | Product size (bp) | Tm (℃) |
| --- | --- | --- | --- |
| mus RUNX2-F | AGTCCCAACTTCCTGTGCT | 19 | 56.32 |
| mus RUNX2-R | GGTGAAACTCTTGCCTCGTC | 20 | 56.31 |
| mus RIP1-F | AAAGGAAACGAAGGGTC | 17 | 48.69 |
| mus RIP1-R | GGCTTAGATTTGGCGGATA | 19 | 51.79 |
| mus RIP3-F | GGGAGATGGAAGACACGG | 18 | 55.61 |
| mus RIP3-R | AGGCAGTAGTTCTTGGTGG | 19 | 54.44 |
| mus MLKL-F | TCGATTCTCCCAACATCTTGC | 21 | 59.80 |
| mus MLKL-R | GTGTAGCCTGTATAAGCCTCTG | 22 | 59.40 |
| mus β-actin-F | GCCTCACTGTCCACCTTCCA | 20 | 63.70 |
| mus β-actin-R | CGCAGCTCAGTAACAGTCCG | 20 | 62.30 |


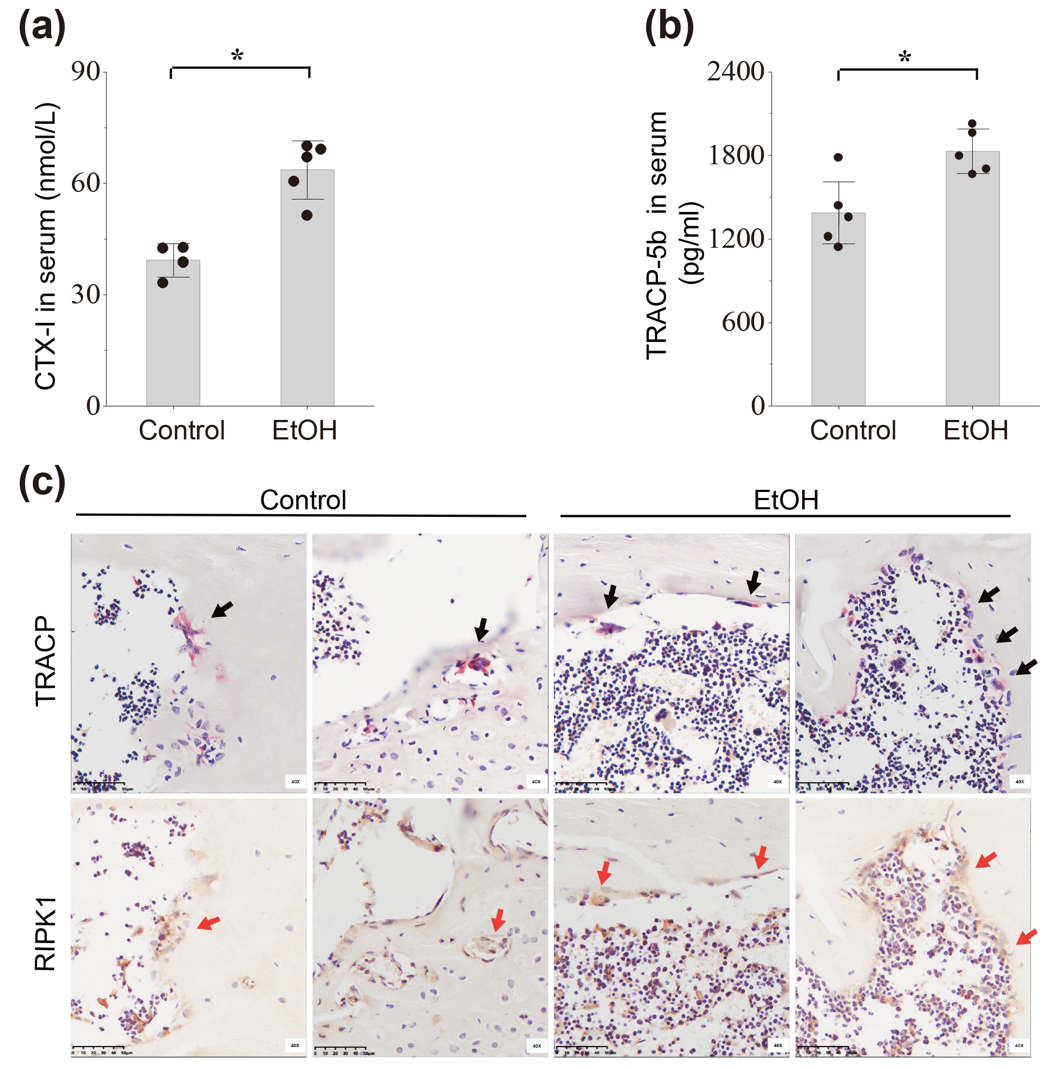


**FIGURE S1** Bone resorption in alcohol-induced osteopenia. (a, b) The serum CTX-I and TRACP-5b concentration of the mice increased in the EtOH-treated group when sacrificed. (c) TRACP staining to identify osteoclasts in the femurs of mice (black arrow) and IHC staining to evaluate the changes in RIPK1 expression (red arrow), finally located the expression of RIPK1 on osteoclasts with the corresponding position. There was no significant difference in the expression of RIPK1 in osteoclasts between the alcohol intervention group and the control group. All experimental data verified in at least three independent experiments. Error bars represent the SD from the mean values. *, p < 0.05. Abbreviations: CTX-I, C-terminal telopeptide of type I collagen; TRACP-5b, tartrate-resistant acid phosphatase 5b.

**
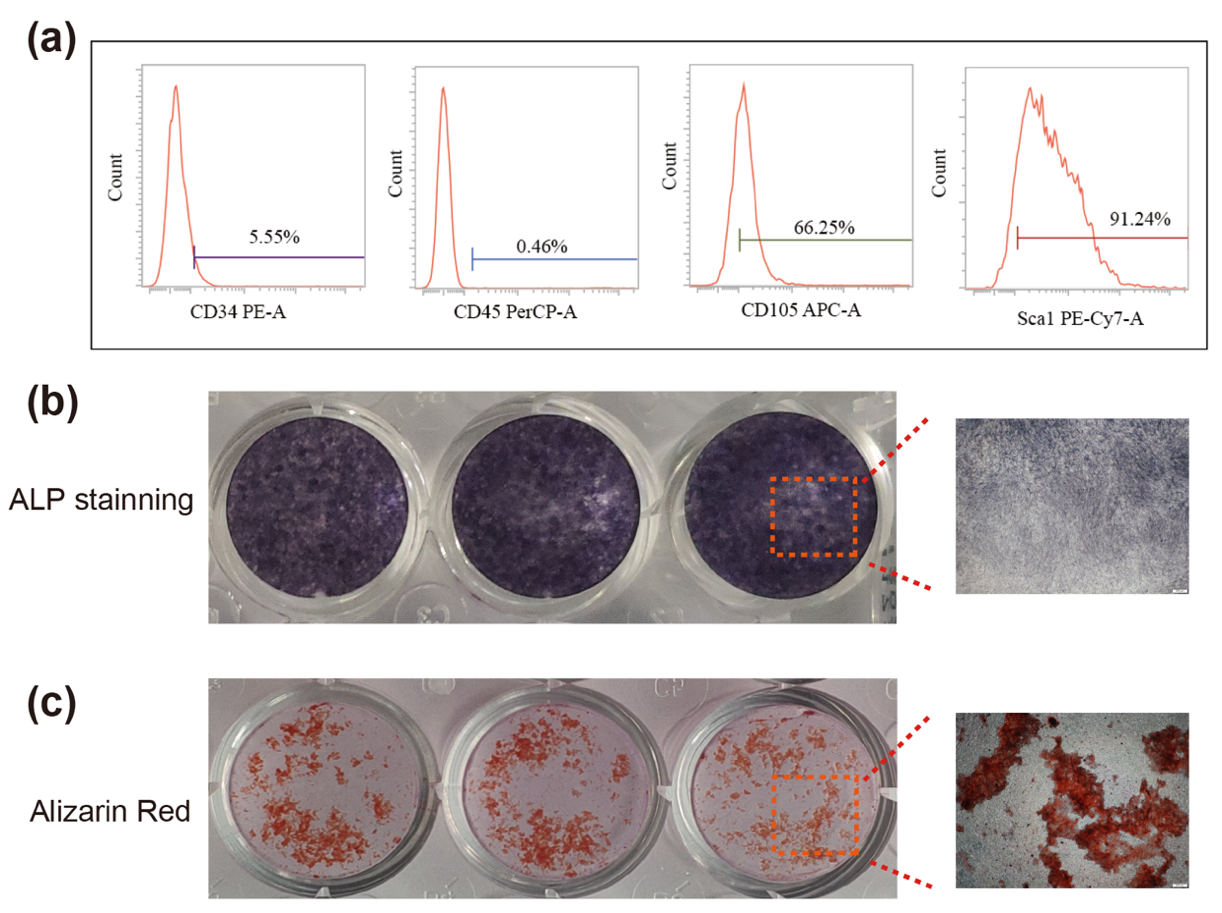
**

**FIGURE S2** Identified the isolated mouse BMMSCs and osteoblast. (a) Flow cytometry analysis of surface markers expression of BMMSCs. The inner part of the red curve indicated the expression levels of BMMSCs surface markers, the results demonstrated that cells were positive for the known BMMSCs markers CD105 (66.25%) and Sca1 (91.24%), but negative for CD34 (5.55%) and CD45 (0.46%). (b, c) Identification of osteoblasts induced by osteogenic differentiation. (b) ALP staining after 7 days of osteogenic differentiation to evaluate alkaline phosphatase activity of osteoblasts. Three plates represented three experiments respectively. (c) Alizarin red staining after culturing in osteogenic medium for 28 days to identify the formation of mineralized nodules of osteoblasts. Three plates represented three experiments respectively. All experimental data verified in at least three independent experiments.

**
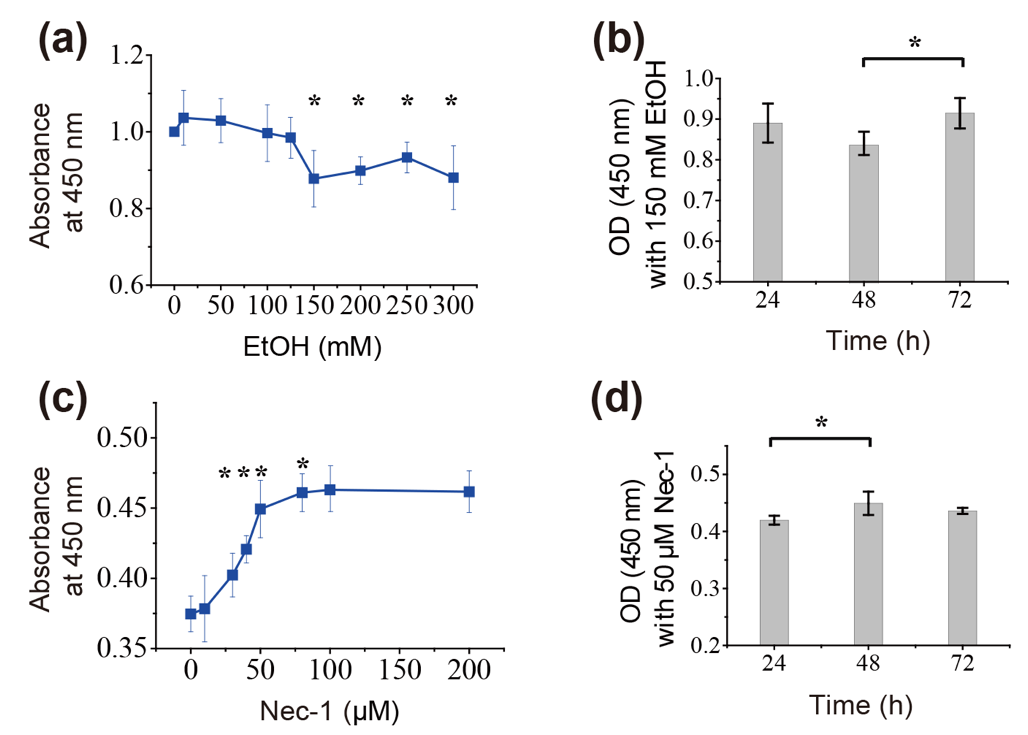
**

**FIGURE S3** CCK-8 assay was performed to determine the cell proliferation ability. (a, b) CCK-8 assay was performed on osteoblasts with EtOH treatment. (a) The cells were treated as indicated above, and the cell proliferation ability significantly decreased when the EtOH concentration is 150 mM. (b) Osteoblasts treated with 150 mM EtOH for 24h, 48h and 72h. (c, d) The cells were treated with Nec-1 after 150 mM EtOH intervention. (c) A CCK-8 assay was performed on osteoblasts treated with 0, 10, 30, 40, 50, 80, 100 and 200 µM Nec-1 for 48 h. (d) The cell proliferation ability was determined with 50 μM of Nec-1 treatment for 24 h, 48 h and 72 h. *, p < 0.05 compared with the blank group. Data are shown as mean ± standard deviation, n = 5. Abbreviations: CCK-8 assay, cell counting kit-8 assay; OD, optical density; h, hour; Nec-1, necrostatin-1.


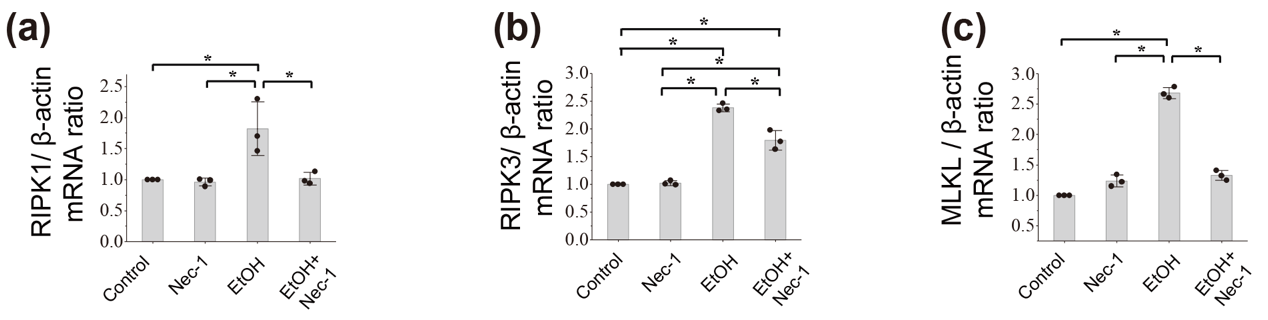


**FIGURE S4** Necrostatin-1 treatment inhibited the RIPK1/RIPK3/MLKL signaling. RT-PCR showed that necrostatin-1 treatment downregulated the increased expression of RIPK1 (c), RIPK3 (d) and MLKL (e) in EtOH-treated MC3T3-E1 cells. All experimental data verified in at least three independent experiments. Error bars represent the SD from the mean values. *, p < 0.05.
